# Understanding the metabolism of the tetralin degrader *Sphingopyxis granuli* strain TFA through genome-scale metabolic modelling

**DOI:** 10.1038/s41598-020-65258-9

**Published:** 2020-05-26

**Authors:** Inmaculada García-Romero, Juan Nogales, Eduardo Díaz, Eduardo Santero, Belén Floriano

**Affiliations:** 1 Centro Andaluz de Biología del Desarrollo, CSIC-Universidad Pablo de Olavide, ES-41013 Seville, Spain; 2 Department of Systems Biology, Centro Nacional de Biotecnología, Consejo Superior de Investigaciones Científicas (CNB-CSIC), 28049 Madrid, Spain; 3 Interdisciplinary Platform for Sustainable Plastics towards a Circular Economy‐Spanish National Research Council (SusPlast‐CSIC), Madrid, Spain; 4 Department of Microbial and Plant Biotechnology. Centro de Investigaciones Biológicas, Consejo Superior de Investigaciones Científicas (CIB-CSIC), 28040 Madrid, Spain; 5 Department of Molecular Biology and Biochemical Engineering. Universidad Pablo de Olavide, ES-41013 Seville, Spain; 6 Present Address: Wellcome-Wolfson Institute for Experimental Medicine, Queen’s University Belfast, Belfast, BT9 7BL United Kingdom

**Keywords:** Bacterial genomics, Bacterial physiology, Bacterial systems biology

## Abstract

*Sphingopyxis granuli* strain TFA is an α-proteobacterium that belongs to the sphingomonads, a group of bacteria well-known for its degradative capabilities and oligotrophic metabolism. Strain TFA is the only bacterium in which the mineralisation of the aromatic pollutant tetralin has been completely characterized at biochemical, genetic, and regulatory levels and the first *Sphingopyxis* characterised as facultative anaerobe. Here we report additional metabolic features of this α-proteobacterium using metabolic modelling and the functional integration of genomic and transcriptomic data. The genome-scale metabolic model (GEM) of strain TFA, which has been manually curated, includes information on 743 genes, 1114 metabolites and 1397 reactions. This represents the largest metabolic model for a member of the *Sphingomonadales* order thus far. The predictive potential of this model was validated against experimentally calculated growth rates on different carbon sources and under different growth conditions, including both aerobic and anaerobic metabolisms. Moreover, new carbon and nitrogen sources were predicted and experimentally validated. The constructed metabolic model was used as a platform for the incorporation of transcriptomic data, generating a more robust and accurate model. *In silico* flux analysis under different metabolic scenarios highlighted the key role of the glyoxylate cycle in the central metabolism of strain TFA.

## Introduction

Members of the *Sphingomonadales* order, which includes the *Sphingomonadaceae* and *Erythrobacteraceae* families, are known as degraders of a large diversity of recalcitrant compounds^1^. Within the *Sphingomonadaceae* family, the sphingomonads group, composed of the ubiquitous genera *Sphingomonas, Sphingobium, Novosphingobium* and *Sphingopyxis*^2^, contains metabolically versatile bacteria capable of using a large number of recalcitrant compounds, mainly aromatic compounds and their derivatives, as sole carbon and energy source^3^. In addition, the genus *Sphingopyxis* includes organisms adapted to oligotrophic environments, which show specific genomic characteristics such as a low number of rRNA operons, prophages or CRISPRs sequences^4^. Moreover, oligotrophs show overrepresentation of Clusters of Orthologous Groups (COGs) for lipid transport and metabolism (I) and secondary metabolite biosynthesis, transport, and catabolism (Q) among others^4^.Thus there is a need to better understand the metabolism of these degradative bacteria and to potentially reveal new insights into their degradative capabilities and lifestyle. To this end, we set out to establish a GEnome-scale metabolic Network REconstruction (GENRE) for a member of this genus that was likely to prove a powerful tool for future studies^5–8^. A GENRE is a database with specific information about the species of interest generated from genomic data, and that contains detailed information about metabolism, the network of reactions, including stoichiometry and reversibility, the Gene-Protein-Reaction (GPR) associations and other available biochemical and physiological data^6,9^. The development of COnstraint-Based Reconstruction and Analysis (COBRA) methods^10^ has allowed the generation of quantitative GEnome-scale metabolic Models (GEMs), that can be used to understand phenotypes in terms of metabolic fluxes. GEMs allow the integration of additional information, such as gene expression data, compartmentalisation of the pathways and probabilistic transcriptional regulation data, resulting in more robust metabolic models with enhanced predictive capabilities. Nowadays there are tools that automatically generate GEMs, like Path2Models^11^, CarveMe^12^ or AGORA^13^. However, manual curation, although tedious and time-consuming, always provides a high-quality and more reliable metabolic model. A recent review of GEMs shows that only 1.9% of the bacterial models have been manually reconstructed^8^. Some applications of well-curated GEMs include the design of microorganisms for the production of chemicals, the prediction of enzyme functions or the discovery of drug targets in pathogens^8^.

According to the NCBI Genome database (Genome List)^14^, 198 and 813 genome sequences of *Erythrobacteraceae* and *Sphingomonadaceae*, respectively, are available. However, despite the environmental significance of these bacterial groups, only one metabolic reconstruction using the genomic information of *Zymomonas mobilis*, *i*EM439, has been generated thus far^15^. This species is a well-known member of the *Sphingomonadaceae* family because of its ability to produce ethanol in a broad pH range, its ethanol tolerance and its use in other biotechnological applications^16^. In this report, we present a genome-scale metabolic model of another *Sphingomonadaceae, Sphingopyxis granuli* strain TFA, a small, rod-shaped, facultative anaerobic, streptomycin-resistant bacterium that possesses the capability to grow using tetralin, a volatile toxic compound that consists of an aromatic and an alicyclic ring, as sole carbon and energy source^17^. The metabolic pathway for tetralin degradation of strain TFA is the only one completely characterized at both biochemical and genetic levels^18^. This pathway, which seems to have been horizontally acquired by TFA, represents a paradigm of how a bicyclic compound can be metabolised with one set of genes mainly involved in the degradation of aromatic compounds. Moreover, the mechanisms involved in the regulation of the expression of the pathway structural and regulatory genes at both transcriptional and translational levels have been elucidated^18^ showing that genes are induced in the presence of tetralin but subjected to carbon catabolite repression in the presence of preferential carbon sources. The genome of strain TFA has been sequenced, assembled into a single circular DNA molecule and functionally annotated^19^. No free plasmids have been detected in TFA either experimentally or during genome sequencing^19^. In this report, biochemical data and gene annotations have been put together to generate a GEM of strain TFA, that has been manually curated and whose characteristics and predictive potential are presented. New potential carbon and nitrogen sources and metabolic pathways that could support growth of strain TFA have been discovered. Moreover, the metabolic model has been improved by incorporating transcriptomic data obtained under two different growth conditions. Flux Balance Analysis (FBA) has revealed the importance of the glyoxylate cycle in the central metabolism of this bacterium. Overall, the model presented here constitutes the first GEM generated for a member of the *Sphingopyxis* genus and the second within the *Sphingomonadales* order.

## Results

### Characterization of the genome-scale metabolic network in *S. granuli* strain TFA

**Figure 1:**
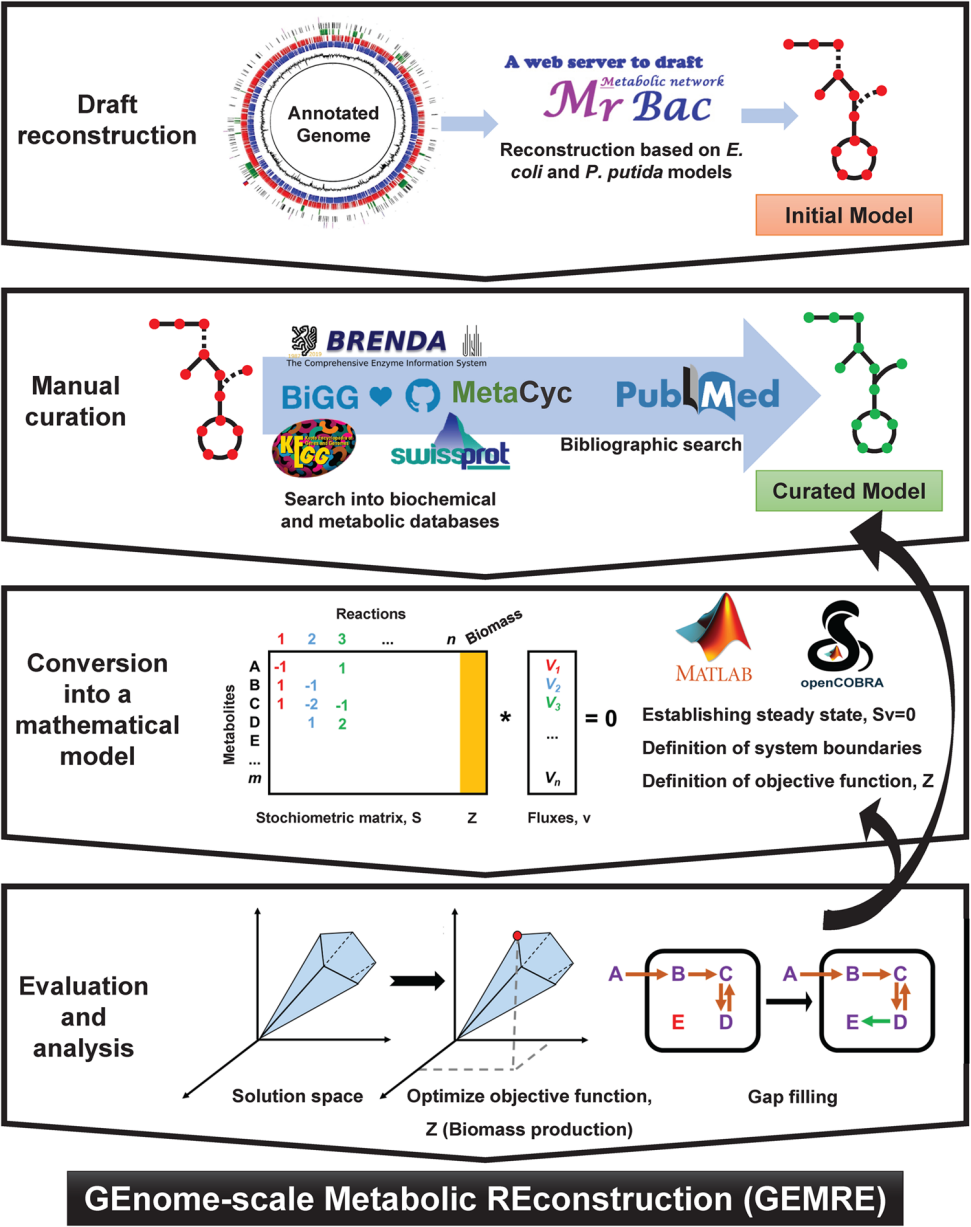
Steps for the construction of the genome-scale metabolic model of TFA *i*IG743. Using the genomic annotation obtained with Sma3s and the *E. coli* and *P. putida* models, the initial reconstruction of the TFA metabolic model was obtained using the server GEMSiRV-MrBac. An exhaustive manual curation was carried out using bibliographic, biochemical and metabolic databases. The curated model was converted into a mathematical model using the COBRA package. Biomass production was the reaction used to evaluate the model and to identify and fill the possible gaps.

The genome-scale metabolic model of strain TFA, called *i*IG743, was constructed following a procedure that includes four steps (Fig. 1). Firstly, an initial draft model based on the *Escherichia coli* K12 model *i*JO1366^20^ and the *Pseudomonas putida* KT2440 model *i*JN1411^21^ was constructed (see Methods). It contained 810 reactions, classified into 84 functional subsystems, 952 metabolites and 386 genes. In this initial draft, 260 reactions and 407 metabolites were added indistinctly from *i*JN1411 or *i*JO1366 based on orthologous genes for those reactions present in both models and in the TFA genome. Interestingly, 410 reactions and 421 metabolites were assigned exclusively from *i*JN1411 whereas only 140 reactions and 124 metabolites came from *i*JO1366. In total, 670 reactions and 828 metabolites were common between *i*JN1411 and the initial TFA draft, whilst 400 reactions and 531 metabolites were shared with *i*JO1366. The higher number of reactions, genes and metabolites taken from *i*JN1411 suggests that TFA is metabolically more similar to *P. putida* KT2440 than to *E. coli* K12 (Supplementary Fig. S1).

Secondly, an exhaustive revision and manual curation of reactions was carried out based on the careful analysis of each GPR included in the draft (see Methods). Therefore, each individual GPR was evaluated based not only on sequence identity, but also considering physiological evidences. For instance, the transport reaction 3_4DHBZ1t_pp_ (3,4-dihidroxybenzoate reversible transporter via proton symport) included in the draft from iJN1411 was eliminated since no enzymes for 3,4-dihydroxybenzoate catabolism were found in TFA. Similarly, the transport reactions for 3-hydroxybutyrate (3-HB) and sebacic acid were included as it is known that TFA grows using these fatty acids as sole carbon and energy sources. Then, the initial model was converted into a mathematical representation and was tested for biomass production (a detailed description as to how biomass composition was determined can be found in the methods section). Finally, the network gaps across the metabolic pathways were filled in by adding new reactions based on the information stored in biological databases, scientific literature and in the functional annotation of strain TFA^19^. During the manual curation, known metabolic features of strain TFA, either experimentally tested or inferred from the genome annotation, were introduced into the metabolic network, including the utilization of tetralin as sole carbon and energy source, the synthesis and degradation of poly-hydroxybutyrate (PHB), the production of the compatible solute ectoine, and the biosynthesis of sphingolipids as components of the cell envelope, which is a characteristic of the *Sphingomonadales* order. To model tetralin degradation, all available biochemical and genetic data were used^18^ (Supplementary Fig. S2A). It is worth noting that *i*IG743 is the first bacterial model in which the aerobic degradation pathway of tetralin has been included providing a platform for further studies on its optimization and integration into bacterial metabolism. For the modelling of PHB synthesis and degradation, the corresponding genes of strain TFA were incorporated into pathways similar to those described for *Ralstonia eutropha* and other bacteria in which the monomer (R)-3-hydroxybutyryl-CoA originates from two molecules of acetyl-CoA via acetoacetyl-CoA^22,23^. Additionally, since strain TFA is able to grow on 3-HB as carbon and energy source, an additional pathway that directly activates 3-HB into its CoA derivative in a reaction catalysed by putative acyl-CoA ligases (SGRAN_2644 or SGRAN_3728) was included (Supplementary Fig. S2B). Genes likely to be involved in ectoine and 5-hydroxyectoine production from *L*-aspartate were located in an operon (*etcABCDask*, SGRAN_2345 to SGRAN_2349) that however lacked a gene for the expected *L*-aspartate-β-semialdehyde dehydrogenase (Asd, SGRAN_0849) (Supplementary Fig. S2C). A second aspartokinase (Ask) encoded by SGRAN_1547 was also included. One of the main characteristics of members of the *Sphingomonadaceae* family is the lack of lipopolysaccharides in their outer membrane, which are replaced by glycosphingolipids^24^. Since sphinganine is part of the central structure of sphingolipids, its synthesis was also included in the model of strain TFA (Supplementary Fig. S2D). Additionally, genes involved in polyvinyl alcohol (PVA) degradation, which show a high similarity with those encoded by the *pva* operon located on a *Sphingopyxis* sp. 113P3 megaplasmid^25^, were annotated in strain TFA’s genome. Thus, the metabolic pathway was reconstructed based on the *Sphingopyxis* sp. 113P3 information (Supplementary Fig. S2E). The identification of a PQQ repeat (Interpro IPR002372, IPR018391) within the amino acid sequence of the putative PVA dehydrogenase of strain TFA (SGRAN_2681) suggests that this enzyme is pyrrolo-quinoline quinone (PQQ)-dependent. However, genes encoding a pathway for the biosynthesis of this cofactor have not been found in the genome of strain TFA. Thus, PVA degradation by strain TFA would need an external supply of PQQ, either directly added to the medium or by being secreted by a syntrophic bacterium as has been described for *Pseudomonas*^26^ and *Sphingomonas*^27^. Although no growth of strain TFA in co-culture with *P. putida* KT2440 and PVA as carbon and energy source has been detected thus far (data not shown), a PVA degradation pathway has been included in *i*IG743.

**Figure 2:**
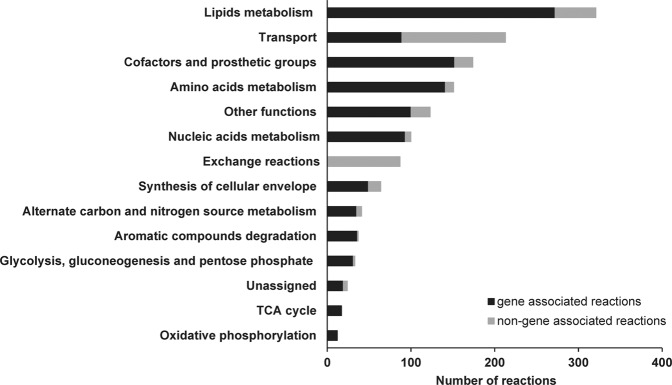
Functional classification of the reactions in *i*IG743. Reactions in the final TFA metabolic model are classified into 115 functional subsystems, which were grouped into the fourteen main categories shown in the figure.

The final model consists of 1397 reactions, 1114 metabolites and 743 genes (Table 1). Compared to other models constructed for α-proteobacteria until date, *i*IG743 contains a large number of reactions and metabolites. Moreover, the manual curation resulted in high or very high (3 or 4) confidence values for 27.8% of the reactions included in *i*IG743. Reactions were classified into fourteen major categories (Fig. 2), of which lipid metabolism is the largest by number of reactions (321 reactions). This model has a large number of reactions for which a gene can be associated (74.9%) although for transport reactions this percentage is lower (58.2%). On the other hand, the exchange reactions are by definition non-gene associated.

**Table 1:** General characteristics of previously published metabolic models for α-proteobacteria and *i*IG743.

Model	Total genes	Genes in the model	TotalRxns	Rxns (genes)	Rxns (no genes)	Metabolites	Year of publication	Reference
*i*EM439 *Z. mobilis*	1823^a^	439 (24%)	692	585	107 (15.5%)	658	2016	^15^
*i*FC579 *N. winogradskyi*	3017^a^	579 (19.2%)	1129	838	291 (25.8%)	1060	2018	^63^
*i*HZ565 *S. meliloti*	6218^a^	565 (9%)	503	481	22 (4.4%)	522	2012	^64^
*i*HZ771 *K. nataicola*	3514	771 (21.9%)	2014	1724	290 (14.4%)	2035	2017	^65^
*i*IG743 *S. granuli*	4190	743 (17.7%)	1397	1046	351 (25.1%)^c^	1114	2020	This work
*i*OR450 *R. etli*	5973^a^	450 (7.5%)	402	339	63 (15.7%)	377	2012	^66^
*i*RP911 *M. extorquens*	6199^a^	911 (14.7%)	1139	936	203 (17.8%)	977	2011	^67^
*i*Rsp1095 *R. sphaeroides*	4410^a^	1095 (24.8%)	1158	1049	109 (9.4%)	1096	2011	^68^
*i*WZ663 *K. vulgare*	3054^a^	663 (21.7%)	830	621	209 (25%)	649	2012	^69^
*i*XW433 *G. oxydans*	2607^a^	433 (16.6%)	859	752	107 (12.5%)	985	2014	^70^
*i*YY1101 *B. diazoefficiens*	8509^a^	1101 (12.9%)	1031	715	316 (30.6%)	766	2017	^71^
Unnamed *M. parvus* OBBP	4845^b^	625^b^ (12.9%)	1324^b^	857^b^	467^b^ (35.5%)	1399	2019	^72^

^a^Data obtained from the Genome List database (https://www.ncbi.nlm.nih.gov/genome/browse/).

^b^Data obtained from https://github.com/SergioBordel/ModelsMethanotrophs.

^c^Including 13 spontaneous reactions.

Only 2.6% of the reactions were assigned to the catabolism of aromatic compounds, such as those involved in the tetralin degradation pathway, the catabolism of the aromatic amino acids phenylalanine, tyrosine and tryptophan, and the degradation pathway of 3-phenylpropanoate and 3-(3-hydroxyphenyl) propanoate. Analysis of essential genes to predict growth on tetralin showed that all the genes identified previously experimentally to be involved in tetralin degradation^18^ (*thn* genes) were essential except *thnN*, *thnO* and *thnP*, which code for a glutaryl-CoA dehydrogenase and an α- and β-flavoprotein subunit, respectively. However, other non-essential genes such as those involved in β-oxidation of pimelic acid (*thnH*, *thnI*, *thnJ* and *thnK*) that are dispensable for growth with tetralin or pimelic acid were not predicted. The large number of β-oxidation pathways and aredundant pimelic semialdehyde dehydrogenase activity (ThnG) found in strain TFA^28^ might explain this discrepancy.

Genes encoding enzymes for the utilization of 3-phenylpropanoate and 3-(3-hydroxyphenyl) propanoate as carbon and energy sources, converting these compounds into pyruvate and acetyl-CoA, were also found^19^ and the corresponding pathways included into *i*IG743. However, no growth of strain TFA was supported by any of these compounds (Table 2).

**Table 2:** Phenotypic validation of TFA growth in minimal medium supplemented with each compound.

Metabolite	*i*IG743 predicted / Experimental		
	C source (N source NH4^+^)	N source (C source β-HB)	C and N source^a^
Amino acids			
D-alanine	+/n.t.	+/n.t.	+/n.t.
D-phenylalanine	+/n.t.	+/n.t.	+/n.t.
D-tyrosine	+/n.t.	+/n.t.	+/n.t.
L-asparagine	+/+	+/n.t.	+/n.t.
L-aspartate	+/n.t.	+/n.t.	+/n.t.
L-glutamine	+/+	+/+	+/+
L-histidine	+/n.t.	+/n.t.	+/n.t.
L-phenylalanine	+/+	+/n.t.	+/n.t.
L-proline	+/+	+/+	+/+
L-tryptophan	+/+	+/n.t.	+/n.t.
L-tyrosine	+/+	+/n.t.	+/n.t.
Dipeptides			
L-alaninylhistidine	+/n.t.	+/n.t.	+/n.t.
L-alaninylleucine	+/n.t.	+/n.t.	+/n.t.
L-alaninylthreonine	+/n.t.	+/n.t.	+/n.t.
L-alaninyltryptophan	+/n.t.	+/n.t.	+/n.t.
β-alanyl-β-alanine	+/n.t.	+/n.t.	+/n.t.
Fatty acids			
Butanoate	+/+	n.t.	n.t.
Hexanoate	+/+	n.t.	n.t.
Octanoate	+/+	n.t.	n.t.
Oleate	+/+	n.t.	n.t.
Pimelate	+/+	n.t.	n.t.
Sebacate (Sebacic acid)	+/+	n.t.	n.t.
3-hydroxybutyrate	+/+	n.t.	n.t.
Aromatic compounds			
3-(3-hydroxyphenyl)propanoate	+/−	n.t.	n.t.
3-phenylpropanoate	+/−	n.t.	n.t.
Tetralin	+/+	n.t.	n.t.
Sugars			
Fructose	+/−	n.t.	n.t.
Glucose	+^b^/−	n.t.	n.t.
Mannose	+/n.t.	n.t.	n.t.
Others			
(R,R)-2,3-butanediol	+/n.t.	n.t.	n.t.
2,5-diketo-D-gluconate	+/n.t.	n.t.	n.t.
2-dehydro-D-gluconate	+/n.t.	n.t.	n.t.
Acetate	+/+	n.t.	n.t.
Agmatine	+/n.t.	+/n.t.	+/n.t.
Coniferol	+/n.t.	n.t.	n.t.
Ethanol	+/−	n.t.	n.t.
Formaldehyde	+/−	n.t.	n.t.
Glycerol	+/n.t.	n.t.	n.t.
L-lactate	+/+	n.t.	n.t.
Polyvinyl alcohol	+^c^/−	n.t.	n.t.
Putrescine	+/n.t.	+/n.t.	+/n.t.

The plus symbol represents either experimentally validated bacterial growth or *i*IG743 growth prediction, whilst minus indicates no growth in experimentally tested carbon sources.

n.t., not tested.

^a^Only amino acids, dipeptides, agmatine and putrescine were tested as nitrogen or carbon and nitrogen sources.

^b^Predicted only if a transporter was added to the model. Since such glucose transport has not been annotated in the genome, it has not been incorporated in the final version of the model.

^c^The model predicts growth only when PQQ is incorporated to the minimal medium.

### Qualitative and quantitative of *i*IG743 validation

To simulate growth of strain TFA in oxic conditions through *i*IG743, a minimal medium was established (see Methods) and the flux through the exchange reaction of each metabolite, including O_2_, was not limited. To test whether *i*IG743 was able to predict cellular growth, the biomass reaction was selected as the objective function and was maximised through the Flux Balance Analysis algorithm (FBA) implemented in the COBRA package. The model was able to predict growth on compounds previously shown to be carbon and energy sources for strain TFA, such as tetralin, 3-HB and sebacic acid, supporting the absence of gaps in the predicted catabolic pathways. Furthermore, the model was able to predict growth in anoxic conditions using nitrate as electron acceptor and 3-HB or sebacic acid as carbon and energy source as had been shown experimentally^19^. To evaluate quantitatively the predictive capability of *i*IG743, the uptake rate of three carbon sources (3-HB, sebacic acid, and L-lactate) for the growth of strain TFA in oxic conditions was experimentally calculated (Table 3; see Methods). Each experimental value of uptake was established as entrance flux in the corresponding exchange reaction and the growth rates were obtained. As shown in Table 3, the average values of the experimentally calculated growth rates of strain TFA with sebacic acid and L-lactate and those predicted by *i*IG743 were in good agreement. However, *i*IG743 predicted significantly faster growth of strain TFA on 3-HB than observed, indicating that adjustments of the model will be required for accurate prediction of growth on this carbon source.

**Table 3:** Uptake rate of different carbon sources experimentally calculated and comparison of the *i*IG743 predicted and experimentally calculated TFA growth rates in minimal medium with each sole carbon source in aerobic conditions.

Carbon source	Experimental uptake rate (mmol·gDW^−1^·h^−1^)	Growth rate (h^−1^)	
		*i*IG743 predicted	Experimentally calculated
Sebacic acid	1.61 ± 0.06	0.29 ± 0.01	0.24 ± 0.01
3-hydroxybutyrate	12.95 ± 1.20	0.85 ± 0.08	0.19 ± 0.00
L-lactate	2.82 ± 0.28	0.12 ± 0.01	0.07 ± 0.00

### Model-based exploration of the metabolic versatility of *S. granuli* strain TFA

Like other members of the genus *Sphingopyxis*, strain TFA is expected to be adapted to oligotrophic environments. Under laboratory conditions, strain TFA grows on a limited number of carbon sources, such as tetralin and its degradation intermediate pimelic acid, 3-HB or sebacic acid. Moreover, ammonia is the preferred nitrogen source and the strain does not grow with nitrate or urea^29^. To explore the metabolic versatility of strain TFA, the complete set of external metabolites present in *i*IG743 was used to identify additional carbon and/or nitrogen sources. As shown in Table 2, model-based analyses identified a wide range of new compounds that strain TFA could use as carbon sources, including mainly fatty acids and amino acids, some of which were experimentally confirmed (Table 2). Additionally, *i*IG743 predicted several amino acids, subsequently confirmed (Table 2), that could be used as sole nitrogen and carbon sources. On the other hand, the model predicted biomass formation with some carbon sources that strain TFA cannot grow on *in vivo*, including ethanol, formaldehyde, 3-phenylpropanoate, 3-(3-hydroxyphenyl) propanoate and fructose. In the case of glucose, the model predicts growth only if a transporter, which has not been annotated in the genome of strain TFA, is artificially included. For growth on PVA, the model predicts that PQQ has to be included as a component of the minimal medium (see above) and shows that for a fixed PVA uptake, PQQ uptake rate determines TFA growth rate on PVA.

When predicted growth rates were normalized according the numbers of carbon atoms present in each carbon source, the model strongly suggested fatty acids as the preferred and more efficient carbon and energy sources over amino acids and other compounds such as lactate or tetralin (Supplementary Fig. S3). Overall these results are in good agreement with the proposed trophic strategy of marine oligotrophic bacteria^4^, where the overrepresentation of genes involved in lipid transport and metabolism in oligotrophic bacteria was related to the higher ATP yield obtained from fats compared to sugars^4^.

### Model-driven analysis of aerobic metabolism in strain TFA

**Figure 3:**
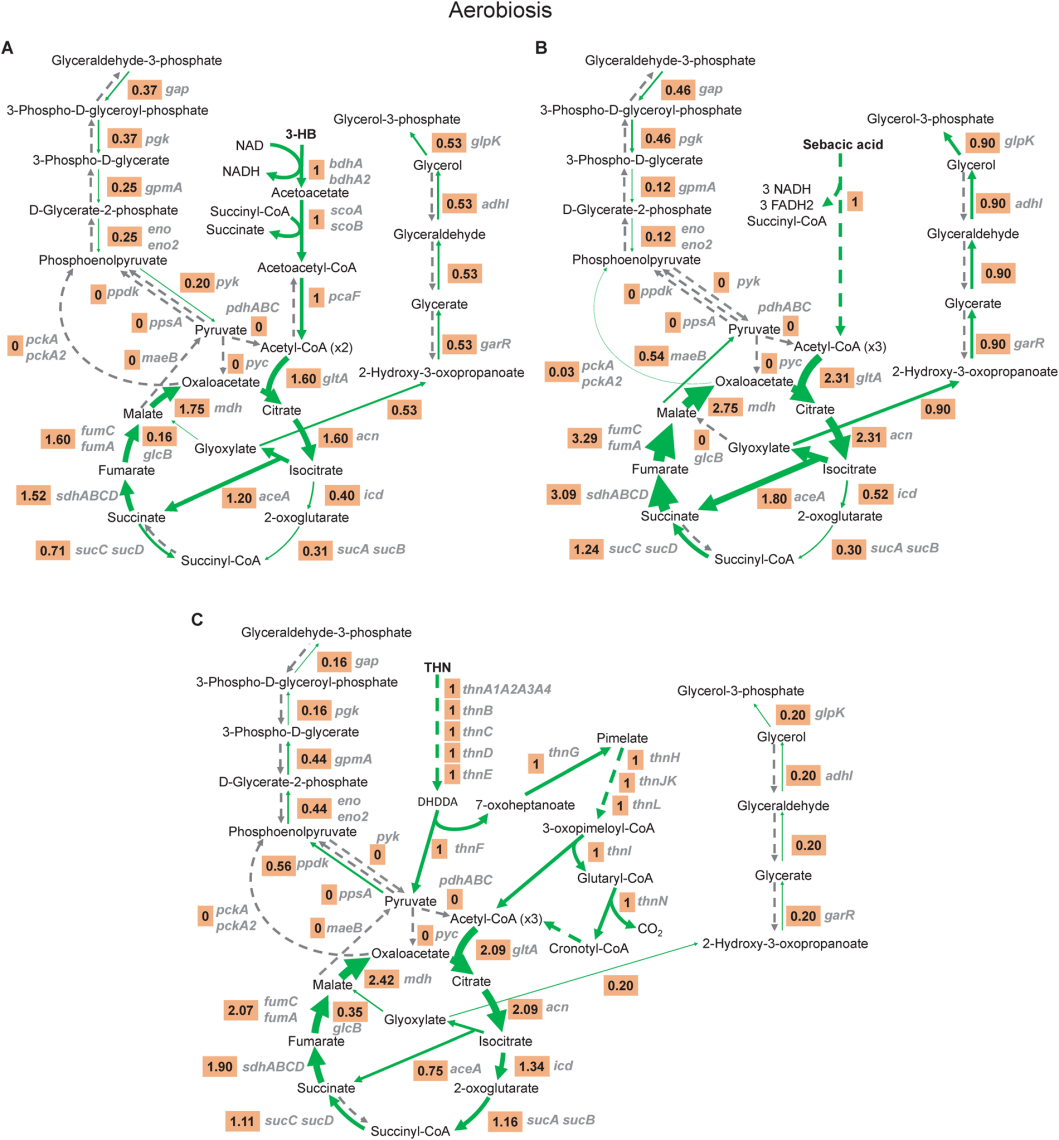
Flux ratio (carbon uptake/reaction flux) through the reactions involved in the catabolism of 3-hydroxybutyrate (**A**) sebacic acid (**B**) and tetralin (**C**). Gene names involved in each reaction are written in grey and the thickness of the green arrows is proportional to the flux ratio. The dashed grey arrows show reactions without flux, dashed green arrows indicate multiple reactions in which intermediates are not represented in the figure.

To analyse the metabolic features of strain TFA when using different carbon sources, we performed a careful flux analysis using *i*IG743 with tetralin, sebacic acid and 3-HB as sole carbon and energy source under oxic conditions (Fig. 3). Since no reliable value for 3-HB and tetralin uptake could be obtained, these uptakes rates (required to constrain the model) were estimated from the experimental growth rates, which were 0.19 h^−1^ for 3-HB (Table 3) and 0.055 h^−1^ for tetralin. The predicted uptake rates were −3 and −0.375 mmol·gDW^−1^·h^−1^ for 3-HB and tetralin, respectively. In the case of sebacic acid, we applied the experimentally calculated uptake rate, −1.61 mmol·gDW^−1^·h^−1^ (Table 3). The analysis of the fluxes showed that the flux through glyoxylate was higher when strain TFA grew on sebacic acid and 3-HB compared to tetralin. In all cases, glyoxylate was directed to glycerol-3-phosphate, although in 3-HB and tetralin its conversion to malate was also predicted. Moreover, the Krebs cycle seemed to operate to provide succinyl-CoA for the activation of acetoacetyl-CoA during 3-HB catabolism (Fig. 3A), while sebacic acid and tetralin could be completely oxidised through this cycle (Fig. 3B,C).

### Model-driven analysis of anaerobic metabolism in strain TFA

Strain TFA is a facultative anaerobe that can grow using either oxygen or nitrate as the terminal electron acceptors^19^. Anaerobic growth of strain TFA was predicted *in silico* using 3-HB or sebacic acid as carbon sources. The value for the experimentally calculated nitrate uptake rate (−2.82 mmol·gDW^−1^·h^−1^, see Methods) was used as constraint in the exchange reactions. The uptake of the rest of the metabolites in the minimal medium was not limited, except O_2_, whose uptake was set to 0. Additionally, since a lower value for the non-growth associated ATP maintenance reaction (NGAM) under anoxic conditions has been described in *E. coli*^30^, NGAM was reduced from 0.92 (aerobic conditions, value obtained from the *i*JN1411 model) to 0.15 mmol·gDW^−1^·h^−1^ to give predicted growth rates of 0.038 h^−1^ on 3-HB and of 0.037 h^−1^ on sebacic acid, while the experimentally calculated values were 0.055 h^−1^ and 0.020 h^−1^, respectively. Concomitant nitrite secretion during anaerobic growth of strain TFA using nitrate as electron acceptor has been experimentally demonstrated^19^. To establish the predictive capacity of *i*IG743, the uptake of nitrate and 3-HB and the excretion of nitrite were simulated by dynamic FBA (dFBA) throughout the cultivation time. For the simulation, the initial concentrations of nitrate and 3-HB were set to 20 mM and 40 mM, respectively, as previously used under experimental conditions^19^, and metabolite uptake rates were set as described above. Initial biomass was experimentally calculated to be 0.038 g·L^−1^.

**Figure 4:**
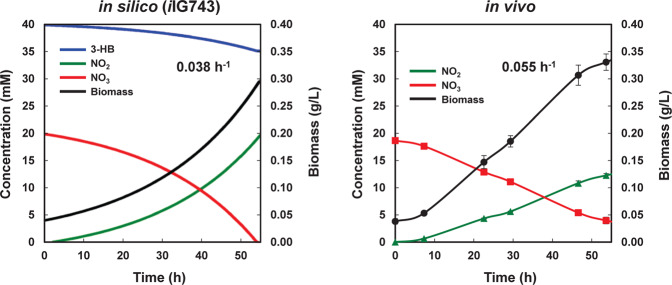
Dynamic validation of TFA under anaerobic growth. Initial concentrations for both *in vivo* and *in silico* growth were set to 40 mM of 3-hydroxybutyrate (3-HB) and 20 mM of nitrate (NO_3_). The left panel shows the combination of the graphs of the simulated growth and metabolite concentrations through the growth curve predicted by *i*IG743 and the right panel the experimental data adapted from García-Romero *et al*.^19^.

As visualized in Fig. 4, nitrate was the limiting compound and an equimolar relation between nitrate uptake and nitrite secretion was predicted and experimentally observed^19^. Similar results were obtained when anaerobic growth with sebacic acid was simulated (data not shown). The results show that *i*IG743 model contains all the information to simulate TFA growth under anoxic conditions.

### Using *i*IG743 as a computational platform for -omics data integration

The predictive capability of a metabolic model for a given condition can lack precision since regulation at the levels of both gene expression and enzymatic activity are not considered, and the fluxes for certain reactions might therefore not reflect physiological behaviour. Consequently, GEMs predictions often benefit from the inclusion of regulatory-derived data such as those obtained by transcriptomics. In addition, beyond providing more accurate predictions, GEMs provide useful computational workflows for the incorporation of -omics data within biological networks^31^. We thus included available transcriptomic data for strain TFA grown aerobically with tetralin and 3-HB^32^ in an attempt to optimize *i*IG743 predictions for the strain grown on each of the two carbon sources. To do this, we reduced the solution space of *i*IG743 by using the GIMME method^33^. The expression of each gene was used as a new constraint in the metabolic model since only genes with a FPKM value higher than the cut-off (see Methods) were considered as expressed under each growth condition. Thus, GIMME constructed condition-specific models by removing reactions encoded by unexpressed genes in each condition and finding a flux distribution consistent with the biological objective. To explore the feasible metabolic states, random sampling was applied to each new model. In this process, the possible fluxes and the probability of each one were determined for all of the reactions in the network, (as visualized in the Supplementary Fig. S4), the range of flux distribution for some reactions involved in the central catabolism was reduced and the probability of their optimal flux was increased after the application of the new constraints (green line).This means that the introduction of transcription data narrows the feasible metabolic states in the conditions analysed suggesting that the flux predictions would be more accurate.

**Figure 5:**
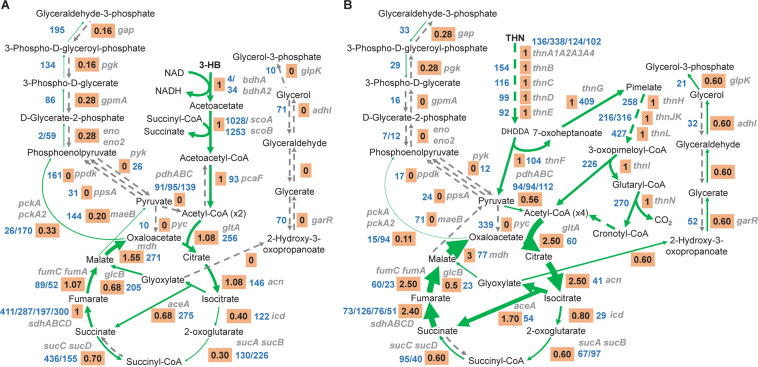
Incorporation of the TFA transcriptomes into *i*IG743. Flux ratio through reactions (orange box) and gene expression (in FPKM, blue) in the pathways involved in the catabolism of (**A**) 3-hydroxybutyrate and (**B**) tetralin. Flux ratio was calculated by dividing the reaction flux by the carbon uptake in the models constrained with expression data. The absence of a FPKM value indicates an orphan reaction without an assigned gene. Arrows thickness is proportional to the flux. The dashed grey arrows show reactions without flux, dashed green arrows indicate multiple reactions in which intermediates are not represented in the figure. Gene names involved in each reaction are written in grey. DHDDA, 2,4-dihydroxydec-2-ene-1,10-dioic acid.

In fact, when introducing the transcription data as the constraint, the predicted fluxes in central metabolic pathways for growth of strain TFA changed significantly (Fig. 5). Thus, with 3-HB, the glycolytic enzymes were directed to gluconeogenesis and supplies of pyruvate and phosphoenolpyruvate were obtained from malate and oxaloacetate, respectively. Moreover, no flux to glycerol-3-phosphate synthesis from glyoxylate was predicted. In the case of tetralin, the inclusion of transcription data resulted in an increase in flux through the glyoxylate cycle and the use of pyruvate to produce acetyl-CoA instead of being used as a source of phosphoenolpyruvate (see Fig. 3C), which was obtained from oxaloacetate. In contrast to 3-HB, an enhancement of glycerol-3-phosphate synthesis from glyoxylate was predicted during growth of strain TFA with tetralin. Moreover, the introduction of transcription data modified the prediction of essential genes. For instance, *aceA* (coding for the isocitrate lyase) was now predicted to be an essential gene for growth on tetralin since glyoxylate became essential for the synthesis of glycerol-3-phosphate as source of glyceraldehyde-3-phosphate (see Fig. 5).

## Discussion

In this work, a genome-based metabolic network of *S. granuli* strain TFA was constructed, successfully validated against some known metabolic states of strain TFA and employed to explore the metabolic versatility of this bacterium. Furthermore, *i*IG743 was used to generate condition-specific models in two well-known metabolic scenarios, growth on tetralin or 3-HB, by adding gene expression data as new constraints. The resulting models showed a significantly better performance, resulting in two unprecedented tools for a system-level study of the metabolism of tetralin and 3-HB in strain TFA. To our knowledge, *i*IG743 is the first genome-scale metabolic model constructed within the sphingomonads group and the second one for the overall *Sphingomonadaceae* family.

Model-based analysis using *i*IG743 successfully identified new metabolites that strain TFA could use as sole carbon and energy source including some fatty acids and amino acids, most of which have been experimentally validated, thus significantly expanding the range of substrates that this oligotrophic strain can use. Interestingly, the model predicted growth with several compounds that experimentally could not support growth of strain TFA. The toxicity of ethanol^34^ and formaldehyde^35^ could explain the incapacity of strain TFA to use these compounds. In the case of glucose, although complete Embden-Meyerhof-Parnas and Entner-Doudoroff pathways were identified, growth was only predicted when a transport reaction was incorporated into the model since no glucose transporter has been annotated in the genome of strain TFA. Moreover, this lack of growth using glucose seems to be characteristic of *S. granuli* species since *S. granuli* Kw07 is also unable to use this sugar in contrast to other *Sphingopyxis* species^36^. Interestingly, the predictions provided by *i*IG743 should assist in the engineering of strain TFA to allow growth on commonly used and cheap carbon sources, expanding the use of strain TFA for a variety of biotechnological purposes.

Other compounds identified as carbon sources by the model were PVA, 3-(3-hydroxyphenyl) propanoate (3HPP) and 3-phenylpropanoate (3PP). Although PVA-supported TFA growth was no detected even in the presence of a PQQ producer, the PVA degradation pathway was included in *i*IG743. The high sequence identity at protein level (>50% with a coverage>85%) and the conservation of the genetic organization of TFA PVA-degradation genes with those present in the well-characterised PVA-degrader *Sphingopyxis* sp strain 113P3 support the existence of that pathway in TFA. However, more experimental assays, such as the use of PVA with different degree of polymerization, are needed since we cannot rule out that those are silent genes. Genes encoding upper pathways (*mhp* and *hca* genes for 3HPP and 3PP, respectively) that converge in 2-oxopent-4-enoate were annotated in the genome of strain TFA. This compound could be further metabolised to pyruvate and acetyl-CoA by the *mhpDEF* gene products. The lack of growth of strain TFA with 3HPP and 3PP could be explained by (i) the absence of appropriate transport systems, (ii) the lack of a functional regulatory system that allowed gene expression, and/or (iii) inaccuracy in gene annotation on enzyme specificity resulting in an incorrectly predicted substrate for a metabolic pathway. The latter could mean that strain TFA might use the predicted pathways to metabolize other similar compounds. Correct substrate prediction is one of the major challenges of automatic GEM generation and requires extensive experimental data. Interestingly, adaptive laboratory experiments (ALE) have shown that some *in silico* predicted phenotypes can be achieved *in vivo* after short-term evolution^37^ by activating putative silent pathways. Alternatively, the presence of these silent metabolic pathways might represent the first step of an evolutionary process to lose the ability to degrade particular aromatic compounds. Consistent with this, it has been proposed that *Sphingopyxis* are specialised to degrade particular compounds rather than exhibiting the ability to utilise a wide range of aromatic substrates^38^.

Although the *in silico* prediction proposed growth with compounds that have been verified *in vivo*, in some cases, such as the use of 3-HB in oxic conditions, *in silico* growth rates were higher than the experimentally calculated. A possible explanation for this discrepancy could be that these compounds were not immediately respired *in vivo* but captured in an intracellular pool of compounds, such as PHB granules, as it has been described in *S. alaskensis* RB2256 during growth with glucose^39^. In fact, 3-HB catabolism implies its conversion into acetoacetyl-CoA and acetyl-CoA (Fig. 3A), which are intermediates in PHB synthesis^40^. Moreover, direct activation of 3-HB with CoA yielding the monomer for PHB synthesis in a reaction catalysed by an acyl-CoA ligase might also take place in strain TFA (Supplementary Fig. S2B). Although PHB synthesis and degradation were incorporated into *i*IG743, all predictions were performed without synthesis of this carbon storage granule. However, it has been reported previously that TFA cells accumulate PHB up to approximately 7% of the total biomass dry weight during growth in minimal medium plus 40 mM 3-HB^41^. As observed in Supplementary Fig. S5A, the demand of PHB in the model leads to a decrease of the growth rate in 3-HB. Therefore, the accumulation of PHB could explain, at least partially, the differences between predicted and measured growth rate in 3-HB.

The demand of PHB synthesis could also explain the discrepancy between the growth rate *in vivo* and the growth rate predicted by the model with sebacic acid under anoxic conditions. Thus, when PHB synthesis was included, the growth rate of strain TFA using sebacic acid as carbon and energy source was more negatively affected than when using 3-HB in anoxic conditions (Supplementary Fig. S5A). Conversely, the same level of reduction was predicted for the growth rate of strain TFA with both compounds under oxic conditions (Supplementary Fig. S5B). Thus, *i*IG743 offers a plausible explanation for these observed discrepancies that will require further experimental analysis of PHB synthesis for confirmation.

*i*IG743 provides an accurate prediction for the growth of strain TFA on tetralin. However, while most of the tetralin degradation genes are predicted to be essential for growth on this compound some of them are dispensable *in vivo*^18^. This incorrect prediction might reflect the inaccurate genome annotation or a broad specificity for some enzymes that cannot be inferred from the annotation. In any case, false positive essential genes are often found in GEM-based analysis. For instance, false positive essential genes were found with the model of *Pseudomonas aeruginosa* strain PA14 (*i*Pau1129)^42^, where the dispensability of *gnyA* for *L*-isoleucine utilization and of *scoA* and *scoB* for growth on 4-hydroxybenzoic acid has been recently demonstrated by Dunhpy *et al*.^43^. In *E. coli*, *aspC*, *argD*, and *gltA* were incorrectly predicted as essential since isoenzyme functions have been described^44^. Thus, as a dynamic process, a genome-scale metabolic reconstruction is expected to undergo continual refinement as inconsistences are found between predictions and experimental data.

As most aromatic compound pathways, tetralin degradation is under carbon catabolite repression in TFA, being *thn* genes repressed in the presence of preferential carbon sources such as 3-HB or sebacic acid^18^. Interestingly, *i*IG743 is able to simulate this phenomenon when oxygen uptake is limited to values predicted by the model when using different carbon sources. For instance, when oxygen uptake was limited to -5 mmol·gDW^-1^·h^-1^, the model discriminates between a good carbon source for TFA, such as 3-HB, and tetralin and establishes a hierarchy of use (Supplementary Fig. S6). Although the involvement of oxygen sensing in carbon catabolite repression in TFA has to be experimentally proven, this is another example of the predictive capability of *i*IG743.

Anaerobic growth of strain TFA using nitrate as electron acceptor could also be modelled using *i*IG743. According to the prediction, nitrate was the limiting substrate for biomass formation and the concomitant reduction of nitrate to nitrite was observed. When the nitrate concentration was increased from 20 to 40 mM, growth of strain TFA for a longer period and with more biomass formation was predicted (data not shown). However, it is known that *in vivo* strain TFA shows similar growth rates and final biomass accumulations in the presence of 20 or 40 mM nitrate as electron acceptor^19^. This may be due to the inhibitory effect of nitrite concentrations higher than 20 mM in the growth of strain TFA^19^. In fact, the difficulty of predicting the toxicity of metabolic intermediates or final products of pathways is one of the major drawbacks when heterologous pathways are incorporated into the metabolism of a target host^45^.

The incorporation of transcriptomic data into metabolic models was expected to result in a more accurate metabolic scenario by defining which genes will be transcribed under a certain environmental condition, which could push fluxes into some specific pathways or reactions. When expression data were incorporated into the model, pathways of central metabolism were modified in different ways depending on the carbon source. However, in all cases, the central role of the glyoxylate shunt was maintained or even enhanced. This shunt bypasses the Krebs cycle conserving carbon atoms for gluconeogenesis and diminishing the number of electrons funnelled into respiration. It is known that this cycle is upregulated when acetyl-CoA is the direct product of a metabolic pathway^46^, which is the case for tetralin, 3-HB and sebacic acid. Moreover, induction of the glyoxylate shunt by oxidative stress has been shown in *P. aeruginosa*^47^. This could be the case in strain TFA when grown on tetralin since this compound is toxic because of the formation of hydroperoxides in the cell^48^. Moreover, the high demand for oxygen during its degradation, which requires high oxygenase activity, might also lead to the formation of hydrogen peroxide^49^ resulting in the induction of glyoxylate synthesis to deal with the generated oxidative stress^47^.

In conclusion, we have developed and enhanced a powerful tool for further metabolic studies of strain TFA that will allow us to better explore and exploit the unstudied metabolic characteristics of this oligotrophic bacterium.

## Methods

### Microorganism and culture conditions

*Sphingopyxis granuli* strain TFA was used as a model organism for this work^17^. Strain TFA was grown aerobically at 30 °C in MML rich medium and in MM minimal medium^17^ containing an appropriate amount of carbon source (Supplementary Table S1). When testing the compound as a nitrogen or carbon and nitrogen source, ammonium was omitted from the MM. For anaerobic growth, strain TFA was pre-cultured aerobically in MM supplied with the same carbon used for the anaerobic culture. The pre-culture at an OD_600_ of 1 was used to inoculate at an initial OD_600_ of 0.1 in standing stoppered bottles filled to the top with MM containing an adequate concentration of the carbon source. Sodium nitrate was added to a final concentration of 20 mM as the terminal electron acceptor for the anaerobic cultures and both the nitrate and nitrite present in the medium were monitored as described^19^. Streptomycin 50 µg/mL was used as antibiotic selection, since strain TFA is naturally resistant to this compound. Growth was determined by measuring optical density at 600 nm.

### Model reconstruction

The metabolic reconstruction of *S. granuli* strain TFA was done using a 4-step protocol as described by Thiele and Palsson^6^ using the genomic annotation obtained with Sma3s^19^.

Firstly, two initial models of strain TFA were constructed based on *E. coli* K12 and *P. putida* KT2440 models (*i*JO1366 and *i*JN1411, respectively)^20,21^, using the GEMSiRV-MrBac server as described in^50^. Two lists of orthologous genes, one between strain TFA and K12 and another between strain TFA and KT2440 were obtained by Reciprocal Best Hits using BlastP (≥40% identity, ≥80% coverage and e-value less than 10^-20^). These lists and the corresponding model in SBML format were used as input files for the GEMSiRV-MrBac server to obtain two draft models of strain TFA in SBML format which included the reactions present in *i*JO1366 and *i*JN1411 assigned to the orthologous genes identified in TFA. Both drafts were then merged into one model eliminating redundancies.

Secondly, as part of the manual curation, all the gene-protein-reaction (GPR) associations were manually reviewed. The assignment of each gene-protein to a reaction was validated by BLASTP against the Swiss-Prot database^51^ and/or *Pseudomonas* database (http://www.pseudomonas.com/). To increase the number of GPRs, proteins of strain TFA were considered orthologous to the best hit in the BLASTP alignment when the e-value was less than 1e-5, the identity higher than 30% and the coverage of both proteins in the alignment higher than 70%^52^. Additional criteria such as the absence or presence of a proper catabolic pathway were used to eliminate or to include transport reactions for some metabolites. Furthermore, a confidence value (between 1 and 4) was assigned to each reaction, which indicated the level of biological knowledge. This value was established according to Thiele and Palsson^6^ with 4 being the highest level of knowledge about the metabolic reaction. Metabolic/biochemical databases such as KEGG^53^, BRENDA^54^, MetaCyc^55^ and BiGG^56^ were used to find the information for the confidence value assignation. In addition, notes and bibliographic references were associated to some GPRs, which were used, together with the BLASTP result and the confidence value, to evaluate the inclusion of a reaction into the network.

Additionally, pathways for well-known metabolic features of strain TFA were manually added, as well as the biomass reaction, based on the biomass of *i*JN1411^57^ and *i*JO1366. Since experimental data for macromolecular composition were not available for TFA, standard values were considered for the biomass (Supplementary Table S5). Moreover, the stoichiometric coefficients for amino acids, DNA and RNA were determined computationally using the genomic information (Supplementary Tables S6, S7, and S8, respectively). The composition of murein, lipids, inorganic ions and soluble pool were taken from *P. putida* and *E. coli* and both NGAM and GAM values from *P. putida* KT2440 *i*JN1411 (Supplementary Table S2). Since *Sphingomonadaceae* have sphingolipids in its outer membrane instead lipopolysaccharide (LPS), the latter was removed from TFA biomass and the synthesis of sphingolipids was added in the form of the precursor sphinganine (Supplementary Table S2).

A second biomass reaction for anoxic conditions was established, which lacks some metabolites that cannot be synthesised anaerobically (Supplementary Table S2).

Thirdly, the model was converted into a mathematical representation using the function *xls2model* stored in the COBRA package present in the MatLab. Thus, a stoichiometric matrix S was constructed in which each row represented a reaction and each column a metabolite. The components of the matrix were the stoichiometric coefficients of the metabolites used for the reactions. This conversion transformed the model into the SBML format, which was required to perform further analysis using the COBRA package.

Finally, it was confirmed that all reactions were balanced and that all metabolites were annotated with their charge and location ([c], cytosol; [p], periplasm and [e], extracellular space). Furthermore, the synthesis of all biomass components by the metabolic network was evaluated. If any one component was not produced, the corresponding biosynthesis pathway was manually revised to find gaps and to fill them in. In the same way, the reactions within each subsystem were analysed to corroborate that essential metabolites were properly synthetized. When possible, a gene was assigned to the newly added reaction using the criteria described above. Otherwise, it was incorporated as an orphan or spontaneous reaction based on available literature. Other non-gene associated reactions were also added, such as exchange reactions for all the external metabolites, sink and demand reactions and the non-growth ATP maintenance reaction (NGAM), as described by Thiele and Palsson^6^. Finally, the capacity of the final model to predict growth of strain TFA with different carbon sources was checked.

The reactions and metabolites included in the final *i*IG743 model can be found in the Supplementary Tables S3 and S4, respectively.

### Flux Balance Analysis (FBA) and sampling

The metabolic reconstruction of strain TFA consisted of a network of reactions in which fluxes were constrained in several ways. Firstly, the lower and upper fluxes of each reaction were limited. Secondly, the metabolic matrix S was imposing constraint through the stoichiometric coefficients. And, thirdly, the stationary state was considered in the simulation, which is defined by the equation Sv=0, where v represents the fluxes through the reactions^7,20^

The Flux Balanced Analysis (FBA)^58^ was used to evaluate the biomass production (growth prediction) once the biomass reaction was fixed as the objective function (BOF, Biomass Objective Function). The result when executing FBA was the growth rate (h^-1^) predicted under the specified media conditions. All the exchange reactions were sequentially tested as potential carbon, nitrogen, or both carbon and nitrogen source, establishing an uptake (vi) of -10 mmol·gDW^-1^·h^-1^. For each test, the uptake of the rest of nutrients in the defined minimal medium, including oxygen, was set up to -30 ≤ vi ≤ 0 mmol·gDW^1^·h^-1^ to avoid uptake limitation other than the tested carbon and/or nitrogen source. For the carbon sources, the growth rate was further normalized considering the number of carbons of each source.

Furthermore, the concentration of metabolites in the extracellular space and the biomass formation along the cultivation time were analysed through the dynamic FBA (dFBA)^59^ under anoxic conditions. For that, the initial concentration of 3-HB and nitrate were set to 40 and 20 mM and the uptake to -3 and -2.82 mmol·gDW^-1^·h^-1^, respectively. As required for anoxic simulation, the entrance of oxygen was set to 0 mmol·gDW^-1^·h^-1^. The initial biomass was experimentally calculated to be 0.038 grams, the timestep was establish in 1 and the number of steps in 200.

The distribution of feasible fluxes in the condition-specific models was calculated by Markov chain Monte Carlo sampling^60^ implemented in COBRA package^61^.

In all the FBA, dFBA and sampling analyse, a minimal media was defined for both oxic and anoxic conditions in which the uptake of the involved compounds was set to –30 mmol·gDW^-1^·h^-1^ to avoid limitation, except for those for which the uptake was experimentally calculated (Table 3).

### Construction of condition-specific models (constraints applied)

The *in silico* defined media provided new constraints in the metabolic system. These media were simulated by giving a value of flux entrance to the exchange reactions of the metabolites present in the minimal medium (MM) and the carbon source in which the predictions were to be done. To apply more specific constraints to the model in MM plus tetralin and MM plus 3-HB, transcriptomic data were incorporated following the protocol described^31^. A flux of 0 through a reaction was considered when the expression of the associated gene(s), measured in FPKM (Fragments Per Kilobase of transcript per Million mapped reads), was lower than the value of the first quartile of the total FPKM values. However, several cut-offs were analysed around the first quartile value, as recommended^37^.

### Calculation of carbon uptake rates

Concentrations of 3-HB and sebacic acid were measured by GC-MS (gas chromatography–mass spectrometry), establishing an initial concentration of 40 mM and 16 mM, respectively. Briefly, 1 mL of sample collected at different time points during growth was centrifuged at 13000 rpm for 5 minutes. 100 µL of the supernatant were evaporated with nitrogen (50 °C) and resuspended on 100 µL of a 0.5 mg/mL solution of citric acid in pyridine. 200 µl of BSTFA + TMCS (99:1) were added to the sample, and the mixture was stirred for 5 seconds and then incubated at 100 °C for 45 minutes. Finally, 1 µL of sample was injected in a DB-17 column (Agilent) and the areas of the picks corresponding to the carbon source and to citrate, which was used as internal standard, were obtained. For *L*-lactate uptake, a lactate dehydrogenase assay was carried out to determine the concentration of *L*-lactate in the supernatant during cultivation. The initial concentration was set to 53 mM. Briefly, 5 µL of supernatant was added to 100 µL of 2x reaction buffer (600 mM glycine, 800 mM hydrazine, 4.8 mM NAD^+^, pH 9.0) and H_2_O was added to reach 200 µL. 1.5 U L-lactate dehydrogenase (Roche) was used to start the reaction and the formation of NADH was measured in a Tecan fluorimeter after 1 h of incubation at 30 °C. Lactate concentration was calculated based on a standard curve^62^. To express the fluxes in mmol·gDW^-1^·h^-1^, the biomass of strain TFA was experimentally measured in DW (Dry Weight) units. To do that, 100 mL of culture were taken at different times along the growth curve in MM plus the corresponding carbon source, centrifuged for 15 min at 5000 rpm and the pellet resuspended in 2 mL of phosphate buffer (Na_2_HPO_4_·12H_2_O 0.34 mM, KH_2_PO_4_ 0.147 mM) and centrifuged for 5 min at 13000 rpm. Finally, the pellet was dried at 80 °C and weighed on a precision balance.

## Supplementary information

Supplementary Information.

Supplementary Information 2.
