# Supplementary material for: Understanding the metabolism of the tetralin degrader Sphingopyxis granuli strain TFA through genome-scale metabolic modelling
Source: Sci Rep. 2020 May 26;10:8651. doi: 10.1038/s41598-020-65258-9 (PMC7250832; doi:10.1038/s41598-020-65258-9)
Supplement: Supplementary file 1 — Supplementary Information. [file 41598_2020_65258_MOESM1_ESM.pdf]

Understanding the metabolism of the tetralin degrader *Sphingopyxis granuli* strain TFA through genome-scale metabolic modelling

Inmaculada García-Romero[1,6], Juan Nogales[2,3], Eduardo Díaz[4], Eduardo Santero[1] and Belén Floriano[5*]

[1]Centro Andaluz de Biología del Desarrollo, CSIC-Universidad Pablo de Olavide, ES-41013 Seville, Spain.

[2]Department of Systems Biology, Centro Nacional de Biotecnología, Consejo Superior de Investigaciones Científicas (CNB-CSIC), 28049 Madrid, Spain.

[3]Interdisciplinary Platform for Sustainable Plastics towards a Circular Economy-Spanish National Research Council (SusPlast-CSIC), Madrid, Spain.

[4]Department of Microbial and Plant Biotechnology. Centro de Investigaciones Biológicas, Consejo Superior de Investigaciones Científicas (CIB-CSIC), 28040 Madrid, Spain

[5]Department of Molecular Biology and Biochemical Engineering. Universidad Pablo de Olavide, ES-41013 Seville, Spain.

[6]Current address: Wellcome-Wolfson Institute for Experimental Medicine, Queen's University Belfast, Belfast, BT9 7BL, United Kingdom.

[*]Corresponding author. Current mailing address: Universidad Pablo de Olavide, Carretera de Utrera Km 1, 41013-Seville, Spain. Phone: 34-95-4349270. Fax: 34-95-4349813. E-mail: bflopar@upo.es

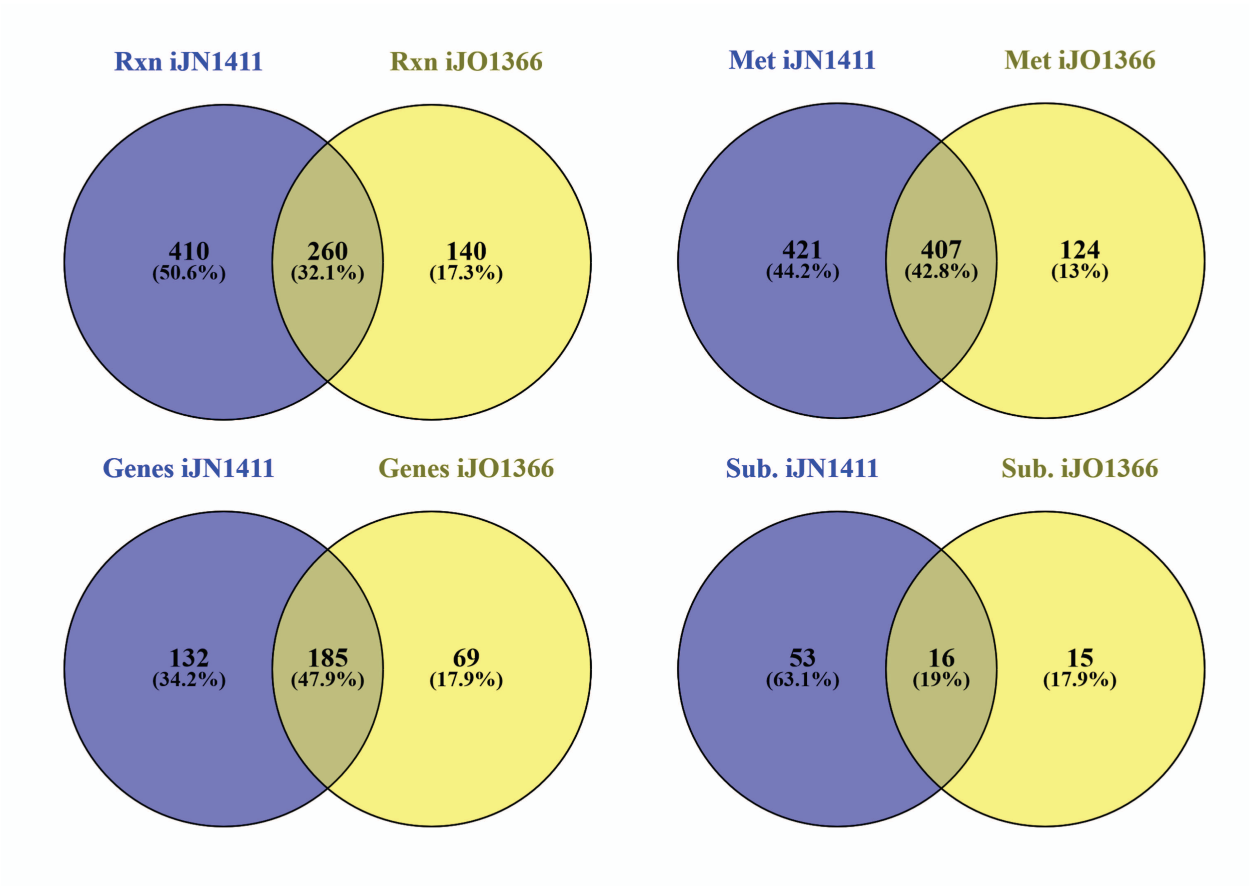

Figure S1. Venn diagrams illustrating the reactions (Rxn), metabolites (Met), genes (Genes) and functional subsystems (Sub) within the initial model draft for TFA metabolism. The elements incorporated into TFA model from *i*JN1411 are represented in blue and those from *i*JO1366 are denoted in yellow.

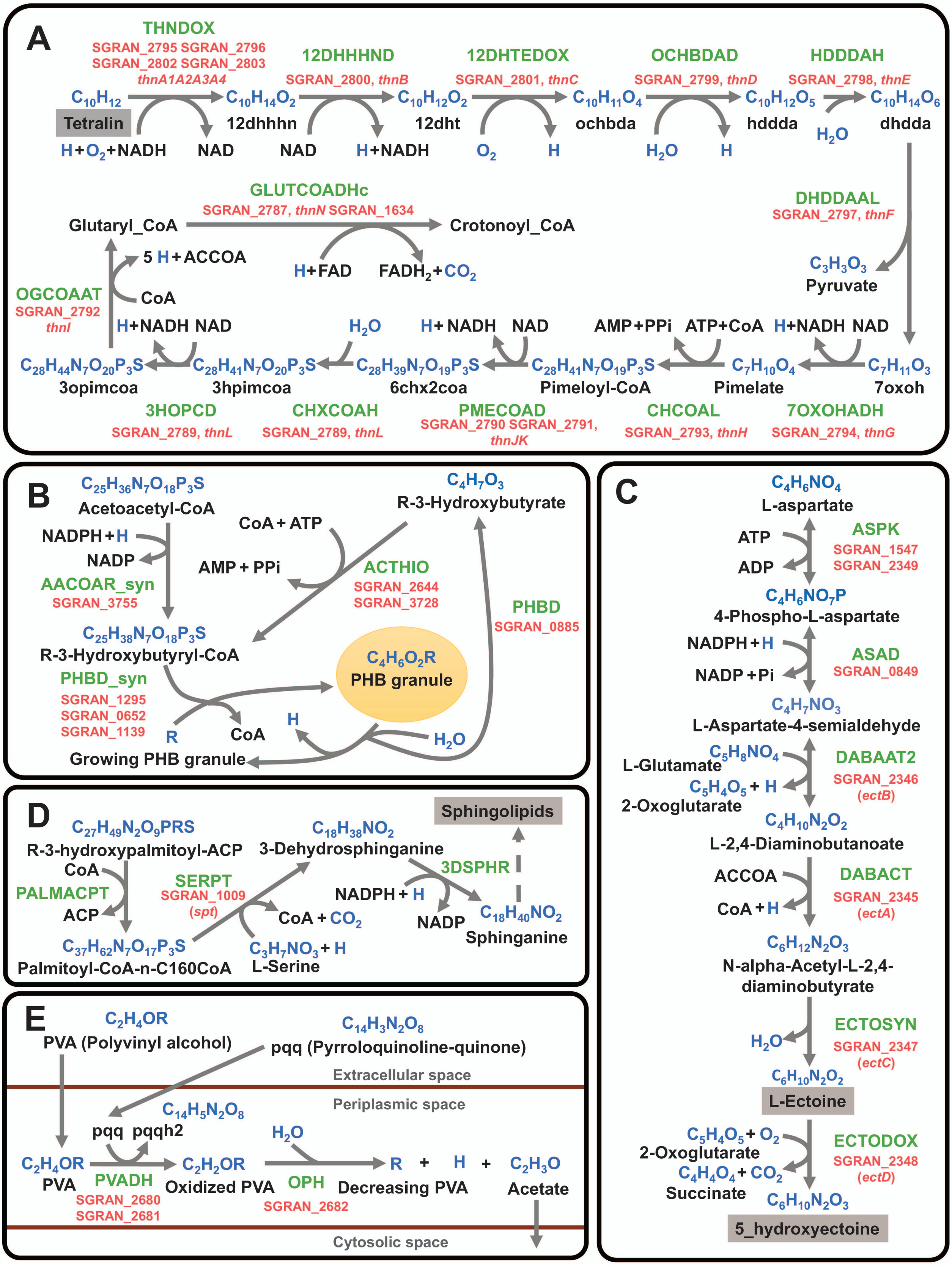

Figure S2. Reconstructed pathways for known metabolic features of TFA incorporated in *i*IG743. (A) Tetralin (THN) degradation pathway, (B) Polyhydroxybutyrate (PHB) polymerization and depolymerization, (C) *L*-ectoine and 5-hydroxyectoine biosynthesis (D)

Sphinganine biosynthesis as precursor metabolite for sphingolipids and (E) Polyvinyl alcohol degradation. The abbreviated name for the reactions is coloured in green and the associated gene in pink. Metabolites names are shown in black and their formula in blue. Some formula has been omitted and some metabolites name abbreviated to simplify the figure. The entire information can be found in the Supplementary Tables S3 and S4 (metabolic reconstruction in excel format).

Carbon source	*i*IG743 Growth rate (h^{-1})
Oleate	**0.1977**
Octanoate	**0.1890**
Hexanoate	**0.1824**
Sebacate (Sebacic acid)	**0.1786**
Butanoate	**0.1691**
L-proline	**0.1631**
3-hydroxybutyrate	**0.1611**
Pimelate	**0.1542**
Tetralin	**0.1536**
L-lactate	**0.1417**
L-glutamine	**0.1416**
L-tyrosine	**0.1240**
Acetate	**0.1238**
L-tryptophan	**0.1200**
L-asparagine	**0.1174**
L-phenylalanine	**0.1174**

Figure S3. Predicted TFA growth rate by *i*IG743 for the sixteen carbon sources validated *in vivo*. Colour scale, from green to red, indicate higher to lower growth rate.

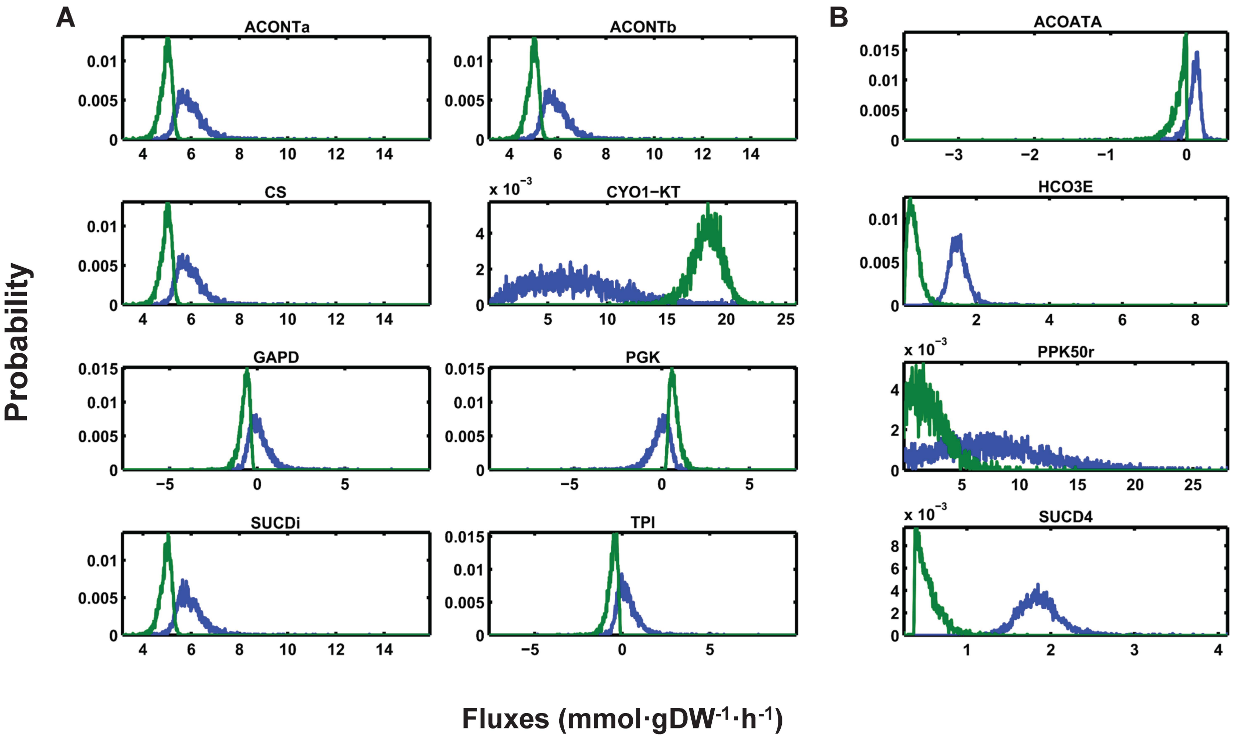

Figure S4. Effect of the incorporation of gene expression constrains on the flux distribution through some important reactions within (A) 3-hydroxybutyrate and (B) tetralin growth conditions modelling. Blue graphs correspond to the unconstraint models and green graphs to the models constrained by expression data. Reactions abbreviation and metabolic processes are: ACONTa, aconitase half reaction A Citrate hydro lyase (TCA cycle), ACONTb, aconitase half reaction B Isocitrate hydro lyase (TCA cycle), CS, citrate synthase (TCA cycle), CYO1-KT, ubiquinol cytochrome *c* reductase (oxidative phosphorylation), GAPD, glyceraldehyde 3 phosphate dehydrogenase (gluconeogenesis), PGK, phosphoglycerate kinase (gluconeogenesis), SUCDi, succinate dehydrogenase irreversible (TCA cycle), TPI, triose phosphate isomerase (gluconeogenesis), ACOATA , Acetyl-CoA ACP transacylase (membrane lipid metabolism), HCO3 equilibration reaction (HCO3), PPK50r, polyphosphate kinase PolyP synthesis (inorganic polyphosphates metabolism) and SUCD4, succinate dehydrogenase (fatty acid metabolism).

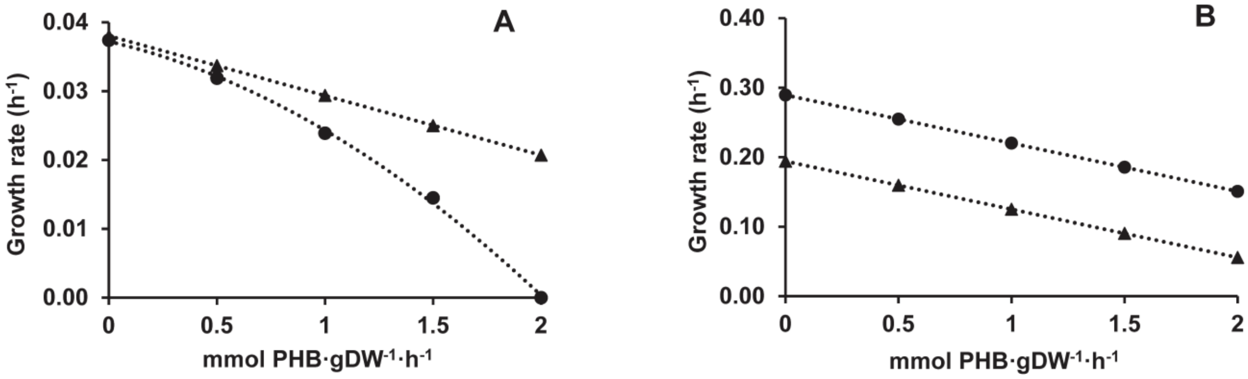

Figure S5. Effect of PHB granule synthesis on TFA growth rates. Growth rates at different PHB synthesis rates were predicted by *i*IG743 model in conditions of anaerobic respiration using nitrate as electron acceptor (A) or aerobic respiration (B) when 3-HB (triangles) or sebacic acid (circles) were used as carbon and energy sources. The uptake of 3-HB and sebacic acid were set at -3 and -1.608 mmol·gDW^{-1}·h^{-1} respectively, in both aerobic and anaerobic conditions. Oxygen uptake was set to 0, nitrate to -2.82 and NGAM reduced to 0.15 mmol·gDW^{-1}·h^{-1} in anaerobic conditions.

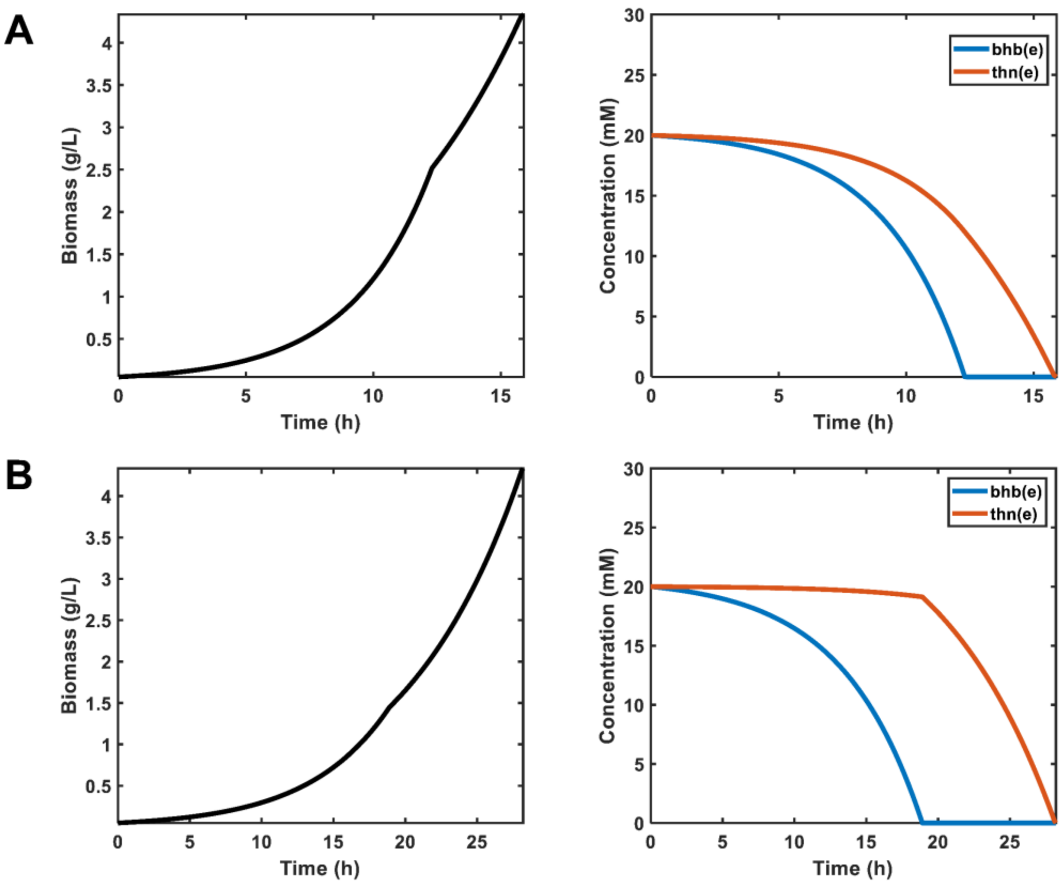

Figure S6. Dynamic simulation of biomass formation (black) and consumption of 3-HB (blue) and tetralin (orange) under no oxygen limitation (A) or reducing the oxygen uptake to -5 mmol·gDW $^{-1}$·h^{-1} (B).
